# A comparison between bronchial blockers and double-lumen tubes for patients undergoing lung resection: A propensity score-matched cohort study

**DOI:** 10.7150/ijms.75835

**Published:** 2022-09-25

**Authors:** Lin Yang, Xiaojin Wei, Bin Wang, Ruping Dai, Feng Xiao, Junmei Xu

**Affiliations:** 1 Department of Anesthesiology, The Second Xiangya Hospital, Central South University, Changsha, Hunan Province, China.; 2 Department of Thoracic Surgery, The Second Xiangya Hospital, Central South University, Changsha, Hunan Province, China.

**Keywords:** bronchial blocker (BB), double-lumen tube (DLT), one-lung ventilation (OLV), lung resection, postoperative recovery, postoperative morbidity, propensity score matching, retrospective study

## Abstract

**Objective:** The aim of this study is to compare the effect of bronchial blockers (BB) and double-lumen tubes (DLT) on patients' postoperative recovery after lung resection.

**Method:** 4,636 patients undergoing lung resection and receiving either BB or DLT intubation were reviewed and matched using the propensity score matching method. The primary outcome was the surgical duration. The secondary outcomes included diagnostic results of postoperative chest X-ray, postoperative oxygenation index, incidence of hypercapnia, hypoxemia and sore throat, chest tube duration, incidence of ICU admission, length of hospital stay and incidence of the 30-day readmission.

**Results:** After matching, 401 patients receiving BB were matched to 3,439 patients receiving DLT. There was no statistical difference on the surgical duration between the two groups (P>0.05). However, compared with the DLT group, patients in the BB group showed more infiltrate especially at the surgery side (14.96% versus 9.07%, P<0.001) based on the chest X-ray, together with higher incidence of ICU admission (5.23% versus 2.61%, P<0.05). Additionally, no statistical differences were found between the two groups about chest tube duration, oxygenation index, incidence of hypercapnia, hypoxemia and sore throat, duration of surgery, hospital stays and 30-day readmission (P>0.05).

**Conclusions:** Compared with the DLT, patients receiving BB technique tend to have increased pulmonary infiltrate (especially the surgery side) and higher incidence of ICU admission at the early post-operative stage, which may have an influence on the patients' recovery.

## Introduction

In most cases, thoracic surgeries, especially video-assisted thoracoscopic surgery, require a well-collapsed lung to facilitate the exposure of the surgical field 1. This goal can be achieved via one-lung ventilation (OLV), a technique that allows ventilation in one lung while leaving the other deflated 2. By far, the most common ventilation strategies for OLV are double-lumen tubes (DLT) and bronchial blockers (BB) 3. DLT is advocated for its quick placement, easy deflation and suction from the isolated lung, and flexible application of continuous positive airway pressure 4. BB, on the other hand, provides minor damage to the trachea probably due to its thinner diameter and lower intra-tracheal pressure, and saves the need for the replacement of the tracheal tube to maintain postoperative mechanical ventilation after surgery 5. At present, the choice of DLT or BB often depends on the preference of the surgeon or anesthesiologist. However, an unsolved question remains as to which is the optimal airway device for performing OLV.

To address this dilemma, researchers have conducted several studies to compare DLT with BB 5-9. A systemic review and meta-analysis 5 conducted in 2015 indicated that DLT is quicker to place and less likely to be incorrectly positioned than BB 5. Instead, BB is associated with fewer incidences of postoperative sore throat, hoarseness, and airway injuries than DLT 6. The similar results were also found in two subsequent randomized controlled trials conducted in 2018 and 2019 7, 8. In comparison, another randomized study conducted by Bussières et al. found that BB enabled faster and better lung collapse than left-sided DLT 9. Nevertheless, the discussion of OLV techniques is ongoing, and there has been no definitive conclusion on which technique is superior.

At present, few studies have compared the postoperative pulmonary complications and the patients' recovery after lung surgery for patients receiving DLT and BB. Understanding the relationship between different OLV techniques and postoperative recovery or complications may aid the clinical decision-making. In this retrospective cohort study, we hypothesized that patients undergoing lung surgery might have different outcomes based on if BBs or DLTs were employed. Because the factors affecting patient outcomes are complex, we use the propensity score matching method to balance the confounding variables and include only lung resection patients to ensure population homogeneity.

## Methods

### Study design

This was a retrospective cohort study approved by the Internal Review Board of the Second Xiangya Hospital, Central South University, Changsha, Hunan Province, China. The electronic medical record system and anesthesia database of the Second Xiangya Hospital were linked, and data was extracted for eligible patients from January 1^st^, 2016 to June 30^th^, 2020. The need for written informed consent was waived due to the retrospective design.

### Participants

Adult patients who underwent lung resection receiving either DLT or BB intubation were eligible for this study. Surgical types included lobectomy, segmental or wedge resection, sleeve resection, pneumonectomy or combinations thereof. The surgeries were performed with an open chest, via thoracoscopy, or with robotic assistance. We excluded patients who underwent bullectomy (the duration of the surgery is too short) or whose surgery involved the trachea or the mediastinum.

### Anesthetic care

All patients received electrocardiogram, heart rate, pulse oxygen saturation, and invasive blood pressure monitoring. Following facemask preoxygenation, anesthesia was induced with midazolam, sufentanil, etomidate, and vecuronium. During the surgery, anesthesia, analgesia and muscle relaxation were achieved by continuous infusions of propofol, remifentanil and cisatracurium, with or without sevoflurane or isoflurane intermittently inhaled.

The tidal volume was approximately set at 8 to 12 ml/kg before and then 6 to 8 ml/kg after the OLV was performed. The respiratory rate was approximately 14 to 20 breaths/min to maintain an end-tidal carbon dioxide of 30 to 40 mmHg. After surgery, the patients were transferred to the post-anesthesia care unit (PACU).

### Intubation and lung isolation

Two types of lung isolation techniques were applied to the patients, i.e., BB (Tappa Medical Technology CO., Hangzhou, China) or DLT (Shiley Endobranchial Tube, Medtronic plc, Minnesota, USA). The choice of BB or DLT was based on the attending anesthesiologist's clinical decision. For patients who received BB, a single-lumen endotracheal tube (SLT, internal diameter of 8.0 mm) was first inserted under a video-laryngoscope after the onset of muscle relaxation and Bispect value fell to 40. The BB was then placed through the SLT guided by a bronchoscope. The attending anesthesiologist confirmed the BB's correct placement and inflated the balloon of the BB with 3-6 mL of air to block the bronchus. For patients who received DLT, the DLT was inserted under a video laryngoscope. Then, the depth of the DLT was adjusted under a bronchoscope and confirmed by the attending anesthesiologist. After the placement of the BB or DLT, the patients were turned to a lateral position. The same attending anesthesiologist rechecked the position of the BB or DLT to ensure correct placement before starting OLV.

For patients who underwent surgery for both sides, either BB or left-sided DLT was used. After the surgery on one lung was finished, the BB would be moved to the other side under the view of a bronchoscope, while for DLT, the ventilation connection would be changed from the bronchial side to the tracheal side before starting OLV.

After the surgery, the BB would be removed from the endotracheal tube; while the DLT would be extubated and another SLT (internal diameter of 7.0 mm for female and 7.5mm for male) was intubated under video laryngoscope.

### Data collection

Data on the patients' age, sex, weight, ASA classification, type of surgery, surgical techniques, results of chest X-ray, duration of the surgery and the chest tube, blood routine, postoperative oxygenation index, incidence of hypoxemia, hypercapnia, sore throat, length of hospital stay, incidence of the ICU admission and 30-day re-admission were extracted. Immediate preoperative and postoperative lab and imaging data were compared. The data collection process was completed by a database analyst who was blinded to the grouping and not involved in the data analysis.

### Outcomes

The primary outcome was the duration of the surgery. Secondary outcomes were: 1) the new infiltrative changes based on the postoperative chest radiographs; 2) postoperative oxygenation index: defined as patients' arterial oxygen partial pressure (PaO_2_) divided by fraction of inspired oxygen (FiO_2_) obtained from the first postoperative blood gas analysis and the medical record in the PACU; 3) postoperative hypercapnia and hypoxemia: defined as arterial carbon dioxide partial pressure (PaCO_2_) >45 mmHg and PaO_2_ <80 mmHg respectively in the PACU when patients woke up and recovered to spontaneous respiration through the tubes; 4) incidence of sore throat: based on whether or not patients woke up and complained about the sore throat; 5) duration of the chest tube; 6) incidence of ICU admission; 7) length of hospital stay; 8) incidence of the 30-day re-admission. A bedside chest X-ray and blood work were regularly conducted at the first day after surgery when patients were in the thoracic department.

### Statistical analysis

No power analysis was performed before the study, and the sample size was based on the available patients who underwent lung resection accepting BB or DLT intubation at the Second Xiangya Hospital from January 1^st^, 2016 to June 30^th^, 2020. Continuous variables were presented as mean ± standard deviation (SD) or median (interquartile range [IQR]), depending on their distribution. The normality of variable distribution was assessed using histograms and Q-Q plots. The categorical variables were presented as numbers and percentages.

Propensity score matching was conducted to minimize the effects of confounders between BB and DLT groups 10, 11. The propensity score was calculated through the multi-variables logistic regression modeling based on covariates including patient's age, sex, weight, ASA classification, surgical techniques. Then, patients were matched on the logit of the propensity using calipers of width equal to 0.2 of the standard deviation of the logit of the propensity score 12. A greedy, nearest-neighbor algorithm was employed to identify candidates and a one-to-eight matching was used to identify pairs, which comprised one patient who received BB and eight patients who received DLT with similar propensity scores. After matching, we calculated standardize differences between the two groups to evaluate the matching effect according to the previous research 13. For both parts, absolute standardize difference <0.1 was considered as well balanced 14.

For normal distributions, including patients' demographics and surgical information, data was analyzed using unpaired t-test; For skewed distributions, including the surgery duration, post-operative oxygenation, chest tube duration and length of hospital stay, data was analyzed using Mann-Whitney U test, followed by independent samples Hodges-Lehman estimator to calculate the median difference and quantify 95% confidence intervals (CI) 15, 16. For categorical data, including the results of chest X-ray, incidences of hypercapnia, hypoxemia, sore throat, ICU admission and 30-days readmission, data was presented as numbers and percentages, and was analyzed using chi-square test; Besides, the effect size was quantified by the odds ratio (OR) and 95% CI using conditional logistic regression 17. Next, due to multiple comparisons and tests, P-values were adjusted to control the familywise error or the false discover rate through Benjamini-Hochberg or Benjamini-Yekutieli procedure 18. And a P-value less than 0.05 (two-sided) were considered statistically significant.

All of the data analysis was performed with Python (version 3.7 https://www.python.org) with its statistical (https://scipy.org) and machine learning packages (https://scikit-learn.org/stable/).

## Results

### Patient characteristics

**Figure 1:**
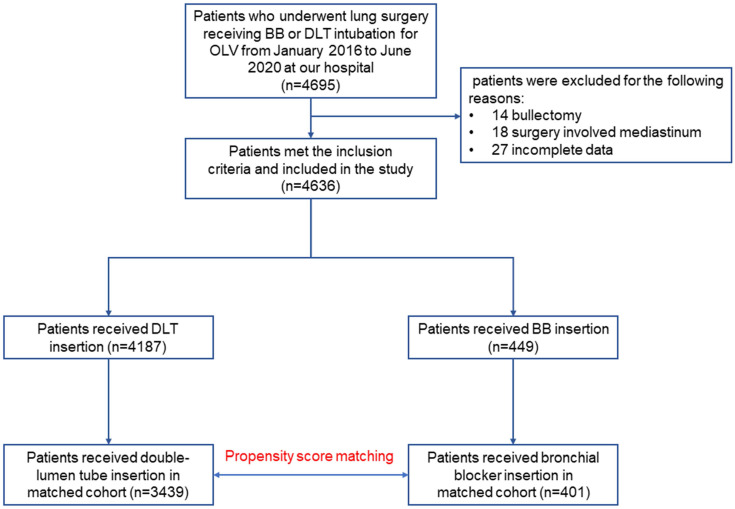
Study flow chart.

Table 1 summarizes the patient demographics and surgery-related information. Data from 4,695 patients who underwent lung resection receiving DLT or BB intubation for OLV were extracted from January 1^st^, 2016 to June 30^th^, 2020. After excluding 59 ineligible patients, we analyzed 4636 patients (Figure 1). Altogether, the mean age and weight were 54.5 years old and 60.4 kg respectively. The proportion of male and female patients were 2,741 (59.12%) and 1895 (40.88%), and the percentage of patients with ASA II was 37.18%. Of all the patients included, 449 (9.7%) patients received BB intubation, while 4187 (90.3%) patients received DLT intubation. Additionally, the most common surgery type was lobectomy, followed by segmental or wedge resection, sleeve resection, and pneumonectomy. Most patients received thoracoscopic surgery, followed by open and robotic techniques.

### Matching

**Figure 2:**
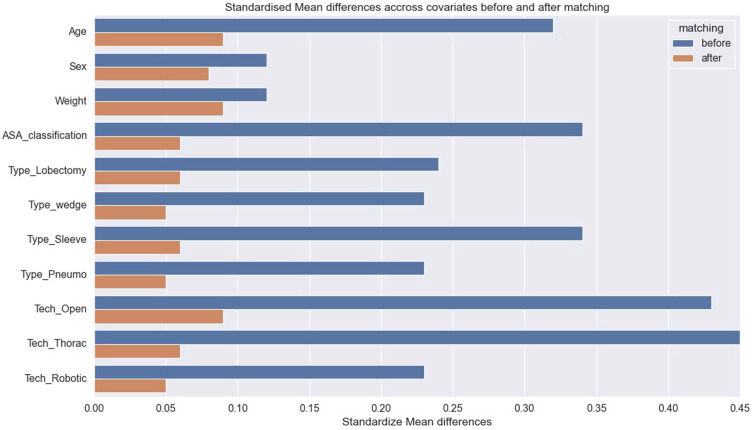
Covariate balance before and after propensity score matching.

Four hundred and one patients who received BB were matched to 3,439 patients who received DLT (Table 1 and Figure 1). In the matched cohort, patients had a mean age of 54.3 years, weight of 60.4 kg. Besides, the percentage of male/female patients were 57.01%/42.99%, and the percentage of patients with ASA II was 37.06%. Following matching, the standardized differences for all the variables were less than 0.1, indicating that the patients who received BB or DLT were well-balanced (Table 1, Fig. 2).

### Results of the primary analysis

As shown in Table 2, after matching, the median of the surgery duration in the BB group was 2.4 hours [1.8, 3.2], which was statistically comparable with the DLT group (2.4 [1.8-3.1], P=0.887).

For secondary outcomes, the percentage of patients in the BB group with no new infiltrate in the chest X-ray was 25.69%, which was statistically lower than the DLT group (32.65%, odds ratio (OR): 0.71, 95% confidence intervals (CI): 0.60 to 0.84, adjusted P=0.017). Meanwhile, at the surgery side, 14.69% of patients in the BB group developed an increasing infiltrative change (<1/3 lung field), which was higher than the DLT group (9.07%, OR: 1.76, 95% CI: 1.37 to 2.27, adjusted P<0.001). Otherwise, no statistical differences were obtained from the comparisons between the two groups at the ventilated side, both sides or >1/3 lung field infiltrate (P>0.05). Besides, patients in the BB group showed higher incidence of ICU admission than the DLT group (5.23% versus 2.61%, OR: 2.05 95% CI: 1.29 to 3.26, adjusted P=0.045). Meanwhile, other parameters, including postoperative oxygenation index (363 [270-448] versus 362 [273-443], median difference: 0, 95% CI: -22 to 22, adjusted P=0.887), incidence of hypercapnia (32.17% versus 27.33%, OR: 1.26, 95% CI: 1.10 to 1.44, adjusted P=0.212), incidence of hypoxemia (20.19% versus 21.43%, OR: 0.92, 95% CI:0.76 to 1.13, adjusted P=0.788), incidence of sore throat (49.37% versus 46.17%, OR: 1.13, 95% CI: 1.10 to 1.17, adjusted P=0.549), duration of the chest tube (86 [67-110] versus 90 [68-112], median difference: 1.13, 95% CI: 1 to 5, adjusted P=0.261), hospital stays (10 [9-11] versus 10 [9-12], median difference: 0, 95% CI: 0 to 0, adjusted P=0.788) and incidence of 30-day readmission (2.74% versus 2.09%, OR: 1.31, 95% CI: 0.70 to 2.46, adjusted P=0.788) showed no statistical differences between the two groups.

### Results of the sensitivity analysis

As shown in Table 3, in the cohort before matching, the duration of the surgery was comparable between BB and DLT group (2.4 [1.7-3.1] versus 2.4 [1.8-3.1], mean difference: 0, 95% CI: 0 to 0, adjusted P=0.808). For secondary outcome, the percentage of patients in the BB group with no new infiltrative changes in the chest X-ray was 24.50%, which was similarly lower than the DLT group (37.35%, OR: 0.54, 95% CI: 0.46 to 0.64, adjusted P<0.001); Besides, at the surgery side, 16.25% patients in the BB group developed <1/3 lung field infiltrative change, which was higher than the DLT group (8.79%, OR:2.02, 95% CI:1.61 to 2.54, adjusted P<0.001). Instead, there were no statistical differences between the two groups at the ventilated side, both sides or >1/3 lung field infiltrate (P>0.05). Besides, the incidence of ICU admission in the BB group was 4.89%, which was also higher than the DLT group (2.45%, OR: 2.04, 95% CI: 1.30 to 3.20, adjusted P=0.027). Meanwhile, no statistical differences were found between the two groups about postoperative oxygenation index, incidence of hypercapnia, incidence of hypoxemia, incidence of sore throat, duration of the chest tube, hospital stays and incidence of 30-day readmission (all adjusted P>0.05). All results above were similar with cohort after matching.

## Discussion

### Summary of the results

This retrospective cohort study analyzed data from 4,695 patients who underwent lung resection at the Second Xiangya Hospital between 2016 and 2020. After propensity score matching, we compared 4,636 patients who received either BB or DLT regarding their postoperative chest radiographs, duration of the surgery and the chest tube, postoperative oxygenation index, incidence of hypercapnia, hypoxemia and sore throat, incidence of ICU admission and 30-day readmission and length of hospital stay. Patients who received BB or DLT were well-balanced after matching. Our results showed that both of the two groups have comparable surgical durations; However, patients who received BB tended to have more severe pulmonary infiltrate (especially at the surgery side) and higher incidence of ICU admission. Otherwise, there were no statistical differences between the two groups in the duration of surgery and chest tube, postoperative oxygenation index, incidence of hypercapnia, hypoxemia and sore throat, length of hospital stays and incidence of 30-day readmission. The results based on the original patient cohort and multivariable analysis (sensitivity analysis) were consistent with the above results.

### Interpretation

Thoracic surgery is associated with a series of postoperative complications 19. Strategies and methods to achieve rapid and enhanced recovery after thoracic surgery have been explored in recent years 20. Understanding the relationship between different perioperative management strategies with unfavorable outcomes after lung surgery is of paramount importance.

OLV plays a crucial role in ensuring smooth and steady lung surgery. The mainstream methods of achieving OLV are using either a DLT or a BB. A great number of studies have put the emphasis on the assessment of the airway and tracheobronchial anatomy before choosing an appropriate method for OLV. It has been reported by a previous meta-analysis that both of the two techniques show a similar quality of lung collapse for patients with normal stature 5. Otherwise, between the two techniques, DLT is the more frequently used device, and can be easy to place only via laryngoscope and auscultation 21-25. However, DLT is more rigid and has a larger diameter, which proves to be difficult or even impossible in patients with airway abnormalities and increases the incidences of airway injuries, postoperative sore throat and even vocal paralysis under an oversized selecting size 21, 26-27. In these cases, placing a BB through the inside of a SLT could be a feasible alternative. A growing body of literature has proven that BB is more advantageous than DLT for difficult airways and significantly reduces the incidences of airway injuries and sore throat 8, 23. However, confirmation of the BB's position in a bronchus needs the guidance of fiberoptic bronchoscope, which takes more time than DLT and puts forward the higher requirements for the anesthesiologists 24, 25. Moreover, the incidence of malposition for BB is much higher particularly in patients accepting the right side-BB intubation 21, 26, 28 and it should also be noted that BB insertion has a risk of bronchial rupture if misapplied 29, or being included in the surgical staple line 30. In the present study, we found that the surgical duration was not different between the two groups, which is similar to the findings of prior studies 5. The result is likely to indicate that both of the two OLV techniques are effective at deflating the lung which needs to be dissected.

Compared with the most inspiring studies investigating the use of BB or DLT by assessing their impact on the incidences or severity of trachea and bronchial injuries including hoarseness, vocal cord lesions, or sore throat after surgery, the speed and extent of recovery and the incidence of pulmonary complications after lung surgery should also be taken into great consideration 30, 31. Notably, Chu et al. 32 reported that compared DLT, thoracic patients accepting BB technique are more likely to experience post-operative pulmonary complications and longer ICU and hospital stays will extend if a BB is used rather than a DLT. The results indicate that BB and DLT may have different clinical outcomes for the recovery of thoracic patients. Unfortunately, they didn't retrieve more detailed laboratory or radio-graphic data.

In this study, patients who received BB or DLT were well balanced regarding the risk factors of age, gender, weight, ASA classification, type and techniques of surgery. We found the following positive outcomes: I. The chest X-ray has proven to be indispensable to identify early postoperative lung complications in most patients after surgery. 31, 34-35 We found for the first time that compared with the DLT, patients in the BB group are more likely to develop an increasing infiltrate especially at the surgery side according to the diagnosis of the chest X-ray. This result should be attributed to the fact that the BB used in the present study has a much thinner inside channel than the DLT. As indicated by previous researches 33, 36-37, the lack of a channel (or a thin channel) inside the BB might fail to provide adequate suction from surgery-side lung, which may cause the residue of the secretion, thus increasing the odds of pulmonary infection; II. the incidence of ICU admission in the BB group are also higher than the DLT group. All results above indicate that patients accepting BB technique might undergo more pulmonary complications and systemic inflammation. However, we did not find any significant differences in the incidence of sore throat or hospital stay between the two groups introduced by previous researches 6-8, 31. The discrepancy might attribute to the basic status of the patients, different proportions of the surgery type, etc. Nevertheless, the impact introduced by BB application needs further careful investigation, as patients with severe vital diseases or infections may not be appropriate for the application of BB.

### Limitations

Our study has several limitations. Firstly, although we used propensity score matching to balance patients receiving BB and DLT, there are likely to have been unknown factors, including conversion from one OLV technique to another, changes of surgery type during the operation and so on, that were not adequately adjusted and might have contributed to the residual confounding; Secondly, there was a large bias towards utilization of DLT instead of BB in our institution. DLT instead of BB would be considered as the primary option especially when the surgery is involved the bronchus or the right lung. Whether or not these specific anatomical characteristics need further careful elucidation; Finally, as the patient's data was retrospectively extracted from the electronic medical record, some critical information, including blood gas disturbances during the operation, incidence of post-operative pneumonia, bronchopleural fistula, hemothorax, superficial surgical site infection, reoperation and so on, was not available due to unstructured recordings. For instance, the lung infiltrative changes were simply a measure of the risk of developing pneumonia, but in fact we don't know what the rate of pneumonia development was. Therefore, future studies that can specifically quantify the incidence of pneumonia, and other pulmonary complications, following each method of OLV could produce even more meaningful results.

## Conclusion

In conclusion, this retrospective study has shown that BB application is associated with more severe lung infiltrate (especially the surgery side) and higher incidence of ICU admission at the early post-operative stage, which may affect the patient's early recovery. Whether the use of BB affect patients' postoperative recovery needs further investigation.

## Figures and Tables

**Table 1:** Characteristics of patients before and after propensity score matching

	Unmatched				Matched			
	Bronchial blocker (n=449) ^a^	Double-lumen tube (n=4187) ^a^	p value	absolute standardize difference	Bronchial blocker (n=401) ^a^	Double-lumen tube (n=3439) ^a^	p value	absolute standardize difference^b^
Age, year	54.5 ± 13.3	54.2 ± 12.6	0.656	0.11	54.6 ± 13.0	53.9 ± 12.4	0.262	0.09
Sex, male	242 (53.89%)	2499 (59.68%)	0.021	0.12	214 (53.37%)	1979 (57.54%)	0.121	0.08
Weight, kg	60.6 ± 10.6	60.7 ± 10.3	0.862	0.12	60.2 ± 10.5	60.4 ± 10.1	0.632	0.09
ASA (II/III), II	188 (41.87%)	1536 (36.68%)	0.035	0.34	153(38.15%)	1271 (36.76%)	0.678	0.06
**Type of surgery, n (%)**								
Lobectomy	346 (77.72%)	3301 (78.83%)		0.24	317 (79.05%)	2700 (78.51%)		0.06
Segmental or wedge resection	83 (18.48%)	698 (16.67%)		0.23	71 (17.71%)	611 (17.77%)		0.05
Sleeve resection	11 (2.45%)	134 (3.2%)		0.34	9 (2.24%)	83 (2.41%)		0.06
Pneumonectomy	9 (1.33%)	54 (1.28%)		0.23	4 (0.99%)	45 (1.30%)		0.05
**Surgical technique, n (%)**								
Open	56 (12.47%)	868 (20.73%)		0.43	48 (11.97%)	506 (14.71%)		0.09
Thoracoscopy	383 (85.30%)	3175 (75.83%)		0.45	345 (86.03%)	2881 (83.77%)		0.06
Robotic	10 (2.22%)	162 (3.87%)		0.23	8 (2.00%)	52 (1.51%)		0.05

^a^ Data are presented as mean ± standard deviation, median [interquartile range], or number (percentage). ^b^ Absolute mean differences < 0.1 was considered as well balanced.

**Table 2:** Outcomes for patients who received double-lumen tube or bronchial blocker based on the propensity score-matched cohort

	Bronchial blocker (n=401) ^a^	Double-lumen tube (n=3439) ^a^	Median difference or odds ratio (95% CI) ^b^	P value ^c^	adjusted P value ^d^
**Primary outcome**					
Duration of surgery, hours	2.4 [1.8-3.2]	2.4 [1.8-3.1]	0 (0 to 0)	0.887	0.887
**Secondary outcomes**					
***Chest X-ray infiltrate area***					
No new lesion	103 (25.69%)	1123 (32.65%)	0.71 (0.60 to 0.84)	0.005	0.017
***<1/3 lung filed***					
Surgery side	60 (14.96%)	312 (9.07%)	1.76 (1.37 to 2.27)	<0.001	<0.001
Ventilated side	23 (5.74%)	116 (3.37%)	1.74 (1.12 to 2.69)	0.024	0.056
both sides	25 (6.23%)	209 (6.08%)	1.02 (0.69 to 1.53)	0.989	1
***>1/3 lung filed***					
Surgery side	105 (26.18%)	936 (27.22%)	0.94 (0.81 to 1.12)	0.703	1
Ventilated side	20 (4.99%)	170 (4.94%)	1 (0.64 to 1.58)	1	1
both sides	65 (16.21%)	573 (16.66%)	0.96 (0.76 to 1.21)	0.812	1
Postoperative oxygenation index	363 [270-448]	362 [273-443]	0 (-22 to 22)	0.883	0.887
Hypercapnia (%)	129 (32.17%)	940 (27.33%)	1.26 (1.10 to 1.44)	0.047	0.212
Hypoxemia (%)	81 (20.19%)	737 (21.43%)	0.92 (0.76 to 1.13)	0.613	0.788
Sore throat (%)	198 (49.37%)	1588 (46.17%)	1.13 (1.10 to 1.17)	0.244	0.549
Chest tube duration, hours	86 [67-110]	90 [68-112]	3 (1 to 5)	0.087	0.261
ICU admission (%)	21 (5.23%)	90 (2.61%)	2.05 (1.29 to 3.26)	0.005	0.045
Hospital stays, days	10 [9-11]	10 [9-12]	0 (0 to 0)	0.576	0.788
30-day readmission (%)	11 (2.74%)	72 (2.09%)	1.31 (0.70 to 2.46)	0.506	0.788

CI, confidence interval. ^a^ Data are presented as median [interquartile range] or number (percentage). ^b^ The median difference and 95% CI were calculated using the Hodges-Lehmann estimator. ^c^ The P value was calculated using the Mann-Whitney test with a stratification adjustment for the matched pairs. ^d^ The P value was adjusted using Benjamini-Hochberg or Benjamini-Yekutieli procedure.

**Table 3:** Outcomes for patients who received double-lumen tube or bronchial blocker based on original cohort

	Bronchial blocker (n=449) ^a^	Double-lumen tube (n=4187) ^a^	Median difference or odds ratio (95% CI) ^b^	P value ^c^	adjusted P value ^d^
**Primary outcome**					
Duration of surgery, hours	2.4 [1.7-3.1]	2.4 [1.8-3.1]	0 (0 to 0)	0.392	0.808
**Secondary outcomes**					
***Chest X-ray infiltrate area***					
No new lesion	110 (24.50%)	1564 (37.35%)	0.54 (0.46 to 0.64)	<0.001	<0.001
<1/3 filed					
Surgery side	73 (16.25%)	368 (8.79%)	2.02 (1.61 to 2.54)	<0.001	<0.001
Ventilated side	24 (5.35%)	180 (4.30%)	1.26 (0.83 to 1.91)	0.364	0.509
both sides	29 (6.46%)	278 (6.64%)	0.97 (0.67 to 1.41)	0.962	0.962
***>1/3 filed***					
Surgery side	119 (26.50%)	997 (23.81%)	1.16 (0.99 to 1.35)	0.226	0.395
Ventilated side	27 (6.01%)	192 (4.59%)	1.33 (0.91 to 1.97)	0.215	0.395
both sides	67 (14.92%)	608 (14.52%)	1.03 (0.82 to1.31)	0.874	0.962
Postoperative oxygenation index	347 [258-445]	352 [264-435]	0 (-20 to 20)	0.887	1
Hypercapnia (%)	148 (32.96%)	1269 (30.30%)	1.13 (1.00 to 1.29)	0.268	0.804
Hypoxemia (%)	95 (21.15%)	882 (21.06%)	1.00 (0.83 to1.20)	1	1
Sore throat (%)	218 (48.55%)	1982 (47.33%)	1.05 (1.02 to 1.09)	0.659	0.988
Chest tube duration, hours	86 [66-110]	90 [67-115]	3 (1 to 6)	0.102	0.459
ICU admission (%)	22 (4.89%)	103 (2.45%)	2.04 (1.30 to 3.20)	0.003	0.027
Hospital stays, days	10 [9-11]	10 [9-12]	0 (0 to 0)	0.499	0.808
30-days readmission (%)	14 (1.79%)	85 (2.03%)	1.55 (0.89 to 2.70)	1	1

CI, confidence interval. ^a^ Data are presented as median [interquartile range] or number (percentage). ^b^ The median difference and 95% CI were calculated using the Hodges-Lehmann estimator. ^c^ The P value was calculated using the Mann-Whitney test or Chi-square test. ^d^ The adjusted P value was calculated using Benjamini-Hochberg or Benjamini-Yekutieli procedure.
